# Letter to the editor: Spine surgery cost reduction at a specialized treatment center. einstein (São Paulo). 2013; 11(1):102-7

**DOI:** 10.1590/S1679-45082013000200024

**Published:** 2013

**Authors:** Andrei Fernandes Joaquim

**Affiliations:** Universidade Estadual de Campinas, Campinas, SP, Brazil

I read with interest the article “Spine surgery cost reduction at a specialized treatment center”^(1)^, which compared the estimated costs of different forms of treatment for spine diseases. The authors showed that costs for patients treated without surgery were lower than those for patients who underwent the procedure, and they concluded that treatment at a specialized center represents a reduction of costs.

We know that treatment options for spine surgeries have increased, many of which are unnecessary and are associated to the form of reimbursement for the surgeon^(2–4)^. Nonetheless, I totally agree with the need for creating specialized centers. Although I also believe that many degenerative diseases with surgical indications could be successfully treated clinically, without any of the complications related to procedures, the paper led me to think that cost decrease was the main goal for treatment in this study. The authors did not describe the patients' clinical evolution or compare the patients with a control group. Therefore, the study design was inadequate to conclude that conservative treatment, despite its lower cost, was more effective for the population.

In addition, the report did not mention the paying entity, which is clearly interested in cost reduction and not in improvement of results. As a poor example, if we compare patients with brain tumor who are treated exclusively with corticoids and those who had surgery, the results concerning costs will favor the nonsurgical group even though the survival of the operated patients is likely to be better.

I believe that specialized centers and qualified professionals would lead to reductions in medical costs of spine surgeries without compromising the results and efficacy of treatment. The study design, unfortunately, was not appropriate for the clinical results obtained. For this reason, it is not possible to analyze the efficacy of clinical treatment.
