# Supplementary material for: Letter to the editor: Spine surgery cost reduction at a specialized treatment center. einstein (São Paulo). 2013; 11(1):102-7
Source: Einstein (Sao Paulo). 2013 Apr-Jun;11(2):265. doi: 10.1590/S1679-45082013000200024 (PMC4872907; doi:10.1590/S1679-45082013000200024)
Supplement: Related texts

# Letter: Author’s Reply

**Authors:** Dan Carai Maia Viola

**Affiliations:** Hospital Israelita Albert Einstein, São Paulo, SP, Brazil

Dear colleague,

In our study, we assessed the total cost of treatment in a cohort of patients with spine diseases. We also compared the estimated total cost for first surgical indication and cost of treatment received by patients at a specialized rehabilitation service. Our objective was not to compare the efficacy and endpoints of patients. Rather, our study compared cost values and stated that treatment at a specialized center can be less expensive.

Surely the question of whether to operate or not is better approached by clinical and cost-effectiveness studies and by evaluation of each case. New studies are evaluating and analyzing outcomes and comparing treatment effectiveness. However, such studies are time consuming, especially because results should be followed up for short, medium, and long periods. Currently, we are following postoperative patients for 18 months, and results are similar for patients treated conservatively and those treated surgically. We are using functional scales, pain scales, and quality of life scales. Although we had a substantial number of patients in the beginning, we have not had enough time to obtain a satisfactory number of patients after 18 months of follow-up, particularly because our project had recently begun. As soon as we gather enough good-quality data, we will publish our results on the efficacy of treatment. We believe that, unlike the treatment of brain tumors with corticoids, conservative treatment for spine diseases has good results when the indications are appropriate.
